# Genetic Variants in Immune Related Genes as Predictors of Responsiveness to BCG Immunotherapy in Metastatic Melanoma Patients

**DOI:** 10.3390/cancers13010091

**Published:** 2020-12-30

**Authors:** Romela Irene Ramos, Misa A. Shaw, Leland Foshag, Stacey L. Stern, Negin Rahimzadeh, David Elashoff, Dave S. B. Hoon

**Affiliations:** 1 Department of Translational Molecular Medicine, John Wayne Cancer Institute, Santa Monica, CA 90404, USA; romela.ramos@providence.org (R.I.R.); mas0151@mix.wvu.edu (M.A.S.); Negin.Rahimzadeh@providence.org (N.R.); 2 Division of Surgical Oncology, John Wayne Cancer Institute, Santa Monica, CA 90404, USA; FoshagL@JWCI.ORG; 3 Department of Biostatistics, John Wayne Cancer Institute, Santa Monica, CA 90404, USA; SternS@JWCI.ORG; 4 Department of Medicine Statistics Core, UCLA School of Medicine, Los Angeles, CA 90024, USA; DElashoff@mednet.ucla.edu

**Keywords:** SNP, Bacille Calmette–Guérin, metastatic melanoma, cancer immunology, adjuvant immunotherapy, innate immunity

## Abstract

**Simple Summary:**

The study objective was to determine if an SNP (single nucleotide polymorphism)-based immune multi-gene panel has the ability to predict adjuvant BCG (Bacillus Calmette–Guérin) immunotherapy responsiveness post-tumor resection in AJCC (American Joint Committee on Cancer) stages III and IV metastatic melanoma patients. A pilot study followed by further verification and control melanoma patient cohorts involving three phase III multicenter clinical trials was used to verify if an immune gene SNP panel could identify if adjuvant BCG therapy correlates with disease outcomes. We found a specific immune gene SNP panel that could identify which patients would respond to adjuvant BCG immunotherapy, but it was not applicable in the control non-immunotherapy treated patients. These studies provide evidence that SNP immune-gene assessment has utility in predicting melanoma patient’s immunotherapy responses to adjuvant BCG immunotherapy.

**Abstract:**

Adjuvant immunotherapy in melanoma patients improves clinical outcomes. However, success is unpredictable due to inherited heterogeneity of immune responses. Inherent immune genes associated with single nucleotide polymorphisms (SNPs) may influence anti-tumor immune responses. We assessed the predictive ability of 26 immune-gene SNPs genomic panels for a clinical response to adjuvant BCG (Bacillus Calmette-Guérin) immunotherapy, using melanoma patient cohorts derived from three phase III multicenter clinical trials: AJCC (American Joint Committee on Cancer) stage IV patients given adjuvant BCG (*pilot cohort*; *n* = 92), AJCC stage III patients given adjuvant BCG (*verification cohort*; *n* = 269), and AJCC stage III patients that are sentinel lymph node (SLN) positive receiving no immunotherapy (*control cohort*; *n* = 80). The SNP panel analysis demonstrated that the responder patient group had an improved disease-free survival (DFS) (hazard ratio [HR] 1.84, 95% CI 1.09–3.13, *p* = 0.021) in the pilot cohort. In the verification cohort, an improved overall survival (OS) (HR 1.67, 95% CI 1.07–2.67, *p* = 0.025) was observed. No significant differences of SNPs were observed in DFS or OS in the control patient cohort. This study demonstrates that SNP immune genes can be utilized as a predictive tool for identifying melanoma patients that are inherently responsive to BCG and potentially other immunotherapies in the future.

## 1. Introduction

Melanoma is an antigenic cancer, whereby activated host anti-tumor immunity has been shown to control tumor progression. Adjuvant immunotherapies that have been used in melanoma clinical trials are: Interferon-α-2b (IFN-α-2b), Interleukin (IL)-2, melanoma cell vaccines, BCG (Bacillus Calmette-Guérin), and immune checkpoint inhibitors (ICI) immunotherapy in post-surgical, disease-free AJCC (American Joint Committee on Cancer) stage III and IV patients [1,2,3,4]. Newer immunotherapies using monoclonal antibodies, such as ICIs, which target against CTLA-4, PD-1, or PD-L1 [5,6,7], have improved overall survival time when used as both adjuvant and neo-adjuvant therapies in AJCC stage III/IV melanoma patients [8,9,10,11]. ICI agents, nivolumab and pembrolizumab therapies, which are less toxic and have better recurrence-free survival rates, are the most effective and current standard of care for the treatment of melanoma patients [1]. However, predicting which patients will respond to these different immunotherapies still remains unknown. In order to improve disease outcomes and reduce unnecessary treatments, personalized therapy for melanoma patients may be needed using an evidence based approach of these patients’ inherited immune status [12]. To date, there are no efficient immune indicators or biomarker tests available to use prior to the initiation of treatment to prospectively identify potential responders to adjuvant immunotherapy. To improve the efficacy of immunotherapies, we need to identify patients that will respond prior to treatment. Our approach to address this issue was to assess retrospectively our multicenter adjuvant clinical trials with long-term clinical follow-up.

BCG, an attenuated live strain of *Mycobacterium bovis*, is an immune-modulating agent used in the treatment of melanoma and bladder cancer patients to train innate immunity [13,14,15]. Several clinical trials have assessed BCG as a post-surgical adjuvant treatment in both early- and late-stage metastatic melanoma [3,16,17,18,19]. Moreover, a recent phase-I/II clinical trial study (NCT01729663) for the CSF470 allogeneic cell vaccine (derived from four cutaneous melanoma cell lines) plus BCG and GM-CSF as adjuvants, revealed an enhanced immune response and a longer distant metastasis-free survival with lower toxicity [20,21]. Past evidence shows that the anti-tumor immunity of BCG can be enhanced through training of the innate immune system [14]. Improved outcomes with BCG have been observed in individual melanoma patients. However, it still remains unknown why some patients respond to treatment while others do not [22,23].

With modern day immunotherapies, it is important to identify how a patient’s immune system will respond before treatment. Single nucleotide polymorphisms (SNPs), which are single base pair alterations located in the human genome, can affect specific immune genes activity particularly those involved in response to cancer and other diseases, thereby accounting for patients’ variable responses to immunotherapy [24,25]. SNPs occur in coding and non-coding regions and can cause changes in biological functions through the expression or modification of key immune genes protein expression and their respective function. There are >5 million (https://ghr.nlm.nih.gov/primer/genomicresearch/snp) SNPs in the human genome. The heterogeneity in patients’ immune responses is due to their inherited genetic profiles. To determine the predictive value of immune SNP genes to BCG immunotherapy in melanoma patients, we assessed three phase III multicenter clinical trials involving melanoma AJCC stage III and IV patients who were rendered disease free by surgery and then were monitored in long-term clinical follow-up. In two of the multicenter clinical trials, patients were given BCG to activate their host innate immunity post-surgery after rendered disease-free [16,17,26]. We hypothesized that SNPs in specific immune-genes are related to BCG activation of the host immune response and could predict the level of effectiveness of adjuvant BCG treatment in melanoma patients. To test our hypothesis, we developed an immunologic panel of 26 SNPs that included cytokines, chemokines, macrophage/monocyte activation markers, dendritic cells marker, and toll-like receptors (TLRs) to assess their ability to predict patients’ responses to adjuvant BCG immunotherapy post-surgery resection [16,17].

Studies have shown that some patients respond more readily to BCG than others [27,28]. Individuals of Asian and European descent, who usually get BCG vaccinations at an early stage in life, are known to have better immune responses to pathogens due to their trained innate immunity after vaccination. In analogous pharmacogenetics studies, immune-related gene SNPs may underlie differences in an individual’s responsiveness to certain immune drugs [29,30]. In this study, we identified key immune related gene variants that are associated with host trained innate immunity correlating with melanoma patients’ disease outcomes.

## 2. Results

### 2.1. Patient Characteristics

**Figure 1:**
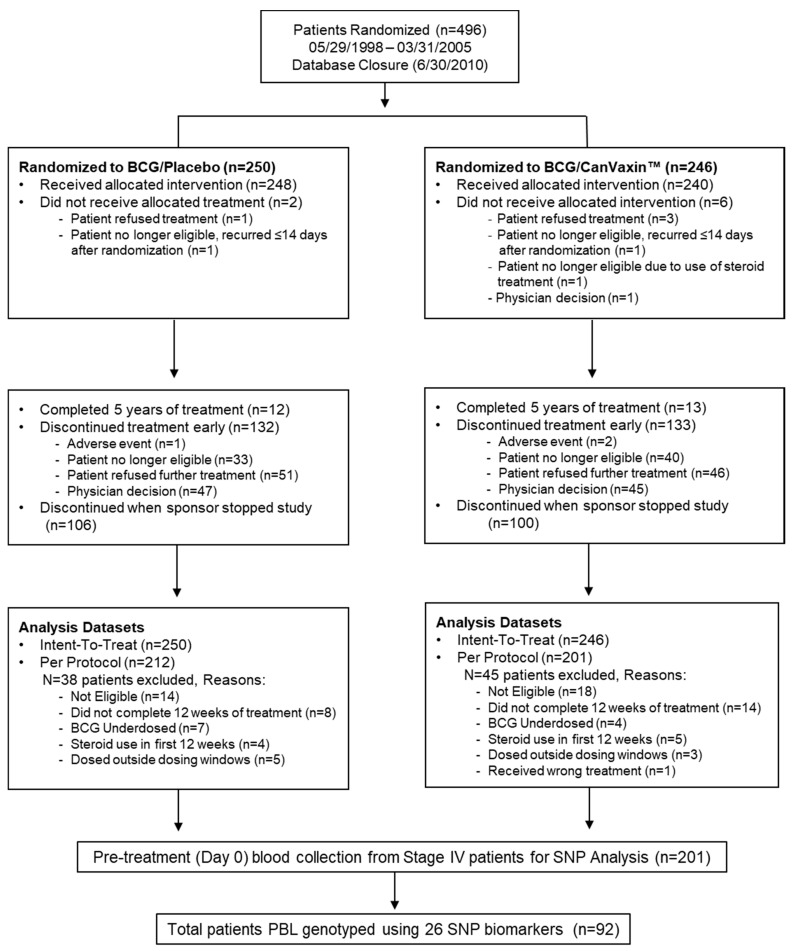
CONSORT diagram of MMAIT-Stage IV patients from the phase III randomized double-blinded international multi-center trial. The flow diagram describing the MMAIT-IV (NCT00052156) trial, is shown. Selected patients (*n* = 92) from MMAIT-IV trial were used as a pilot study cohort. MMAIT = Malignant Melanoma Active Immunotherapy Trial; SLN = Sentinel Lymph Node; PBL = Peripheral Blood Leukocytes; SNP = Single Nucleotide Polymorphism.

**Figure 2:**
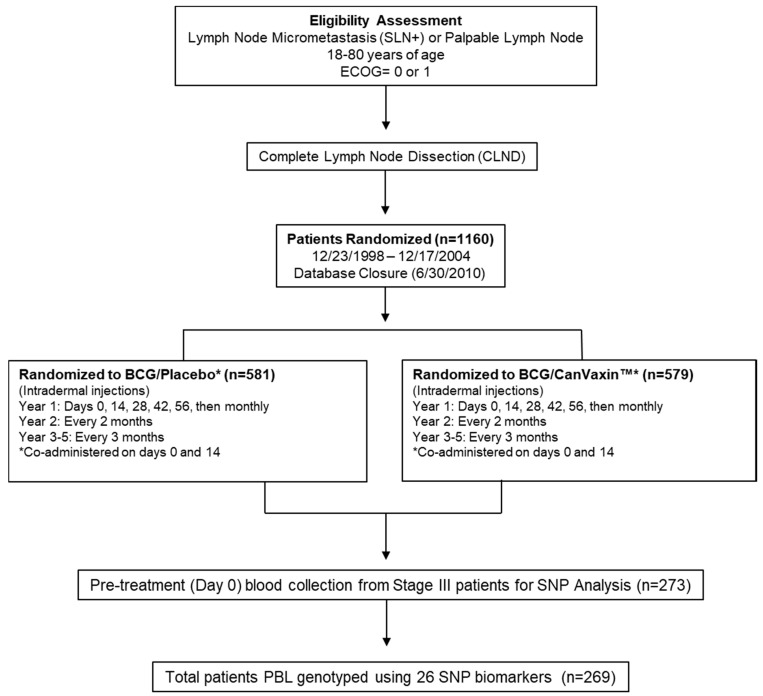
CONSORT diagram of MMAIT-Stage III patients from the phase III randomized double-blinded international multi-center trial. The flow diagram describing the MMAIT-III (NCT00052130) trial is shown. Selected 269 patients from MMAIT-III trial were used as a verification cohort. ECOG = Eastern Cooperative Oncology Group; MMAIT = Malignant Melanoma Active Immunotherapy Trial; SLN = Sentinel Lymph Node; PBL = Peripheral Blood Leukocytes; SNP = Single Nucleotide Polymorphism.

**Figure 3:**
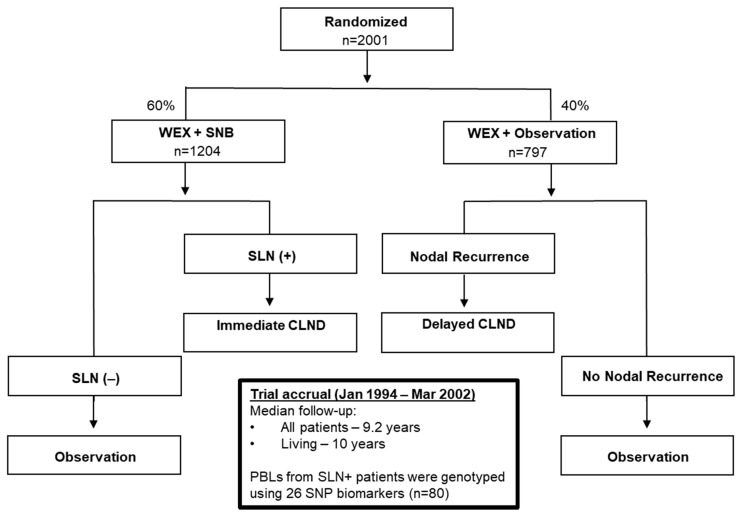
CONSORT diagram of MSLT-I Stage III patients from the phase III randomized double-blinded international multi-center trial. The flow diagram describing the MSLT-I (NCT00275496) trial, is shown. Selected 80 patients from MSLT-I trial were used as a control cohort. MSLT = Multicenter Selective Lymphadenectomy Trial; SLN = Sentinel Lymph Node; WEX = Wide Excision; SLNB = Sentinel Lymph Node Biopsy; CLND = Complete Lymph Node Dissection; PBL = Peripheral Blood Leukocytes; SNP = Single Nucleotide Polymorphism. Blood was taken pre-SLN surgery.

The study involved 441 metastatic melanoma patients.The pilot cohort consists of stage IV BCG-treated patients (*n* = 92) from the JWCI Multicenter Malignant Melanoma Active Immunotherapy Trial (MMAIT-IV) (Figure 1 CONSORT diagram; Table S1A).The verification cohort has stage III BCG-treated patients (*n* = 269) from the MMAIT-III (Figure 2 CONSORT diagram; Table S1B), and the control cohort comprises stage III SLN positive non-BCG and non-immunotherapy treated patients (*n* = 80) from the phase III Multicenter Selective Lymphadenectomy Trial of JWCI (MSLT-I) (Figure 3 CONSORT diagram; Table S1C). The clinicopathologic characteristics of the patients used in this study are presented in Table S1A–C. In the MMAIT stage IV study group, there were 58 patients (63%) with disease recurrence and 31 patients (34%) who expired. In the MMAIT stage III study group, 124 patients (46%) with disease recurrence and 86 patients (32%) who expired. In the MSLT-I stage III comparative study group, 39 patients (49%) had disease recurrence and 30 patients (38%) expired. Patients in the trials were selected based on the availability of PBLs and long-term clinical follow-up.

### 2.2. Immune Gene SNP Selection

In this study, immune-gene SNPs were characterized by a MassARRAY SNP genotyping and selected by going through the available literature. SNPs were selected on the basis of their relation to the host immune responses to BCG, tuberculosis (TB), and trained innate tumor immunity. As shown in Table 1, the SNPs that we selected were all previously reported to be important for host trained innate and BCG treatment adaptive immune responses. The following are the list of genes with their respective SNP sites (in parenthesis): Inflammatory cytokines such as, TNF (rs1800629, rs361525), IL1β (rs1143627, rs1143634, rs16944), IL8 (rs4073), IL10 (rs1800896), IL12β (rs17860508), and IL23R (rs1343151). Chemokine receptor/chemokine: CCR5 (rs1799987) and CXCL12 (rs1801157). TLR such as TLR2 (rs4696480, rs5743708); macrophages/monocytes such as: NRAMP1 (natural resistance-associated macrophage protein 1)/SLC11A1 (solute carrier protein 11A1) (rs17215556, rs17235409, rs17235416, rs34448891, rs3731865). NRAMP1 is a membrane-associated transporter of divalent metal ions associated with macrophages in BCG responses [31]. CD14 (rs2569190, rs2569193) is a monocyte/macrophage differentiation antigen on the surface of myeloid lineage, such as monocytes, macrophages, and dendritic cells (DCs) [32]. CD18 (rs684, rs117989670), also known as β_2_ integrin subunit, is linked to monocyte hematopoiesis regulation [33]. Dendritic cells such as CD209 (Cluster of Differentiation 209) is also known as DC-SIGN (Dendritic Cell-Specific Intercellular adhesion molecule-3-Grabbing Non-integrin) (rs4804803). SP110 (rs3948464, rs1135791) is a nuclear body multiprotein complex and has been linked to *Mycobacterium tuberculosis* resistance [34]. BTNL2 (Butyrophilin Like 2) (rs2076530) is a protein coding gene and has been linked to sarcoidosis [35]. Initially, we assessed a total of 43 SNPs of immune-related genes to BCG and innate immunity: TLR4, TIRAP, IFNgamma, RANTES, CCR2, MCP1 (Monocyte chemoattractant protein), IL12, P2 × 7 (Purinergic receptor P2X7), VDR (Vitamin D Receptor), which were narrowed down to the above 26 immune SNP genes based on their significance as SNP panel signature biomarkers and literature studies.

### 2.3. Assessment of SNP and Disease Outcome Analysis

**Figure 4:**
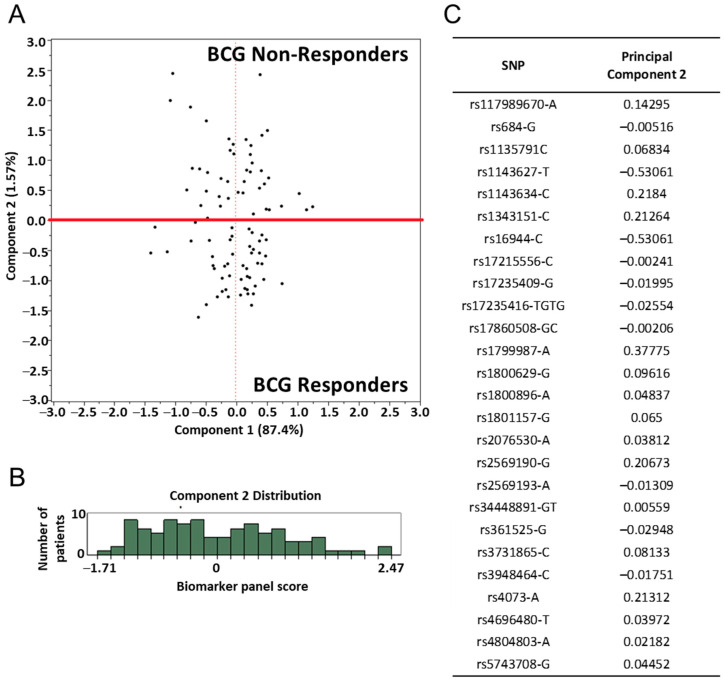
Principle component analysis (PCA) of gene SNP panel in the pilot patient cohort (MMAIT stage IV BCG-treated). (**A**) The gene SNP panel score for each patient is represented as a data point on principal component 1 and 2, representing the majority of the data variability. Red line = cut-off point at 0.005. (**B**) Histograms showing the distribution of principal component 2 among all patients. A cut-off point at component 2 was selected to dichotomize the patients into two groups, termed here as responders and non-responders. (Responders: Comp2 ≤ 0.005; Non-responders: Comp2 > 0.005) The cut-off point was selected at the point of separation in the data distribution. (**C**) The co-efficient for each gene SNP was determined by PCA. The alleles used for calculation of the co-efficient are indicated.

Initially, we assessed the gene SNP panel in Stage IV MMAIT BCG+ placebo and classified patients’ clinical outcomes into “responders” and “non-responders” based on their gene SNP scores. The gene panel score was calculated as described in the Materials and Methods section for each patient. This data was then plotted using Principal Component Analysis (PCA). The variability of the data points is shown on the plotted data in principal component 1 and 2 (Figure 4A). A cut-off point of 0.005 at component 2 was selected to separate responders from non-responders. The distribution of principal component 2 among all patients is shown in a histogram (Figure 4B), wherein the cut-off point selected was based on the separation of the data distributed by principal component 2. The allele used for calculation of the co-efficient for each SNP was determined by PCA and is indicated in Figure 4C.

To further explore the findings, an additional PCA was performed in the same cohort of Stage IV MMAIT BCG+ placebo patients. Plotting principal components 1 and 2 revealed a separation between responders and non-responders (Figure S1A). The said cut-off of 0.005 is shown to dichotomize the patients into responders and non-responders in principal component 1. The distribution of principal component 1 among the 92 patients is shown in a histogram along with the cut-off of 0.005 as a threshold to separate the two groups (Figure S1B). Correspondingly, Figure S1C reports the principal component 1 coefficient for each SNP found by the PCA.

**Figure 5:**
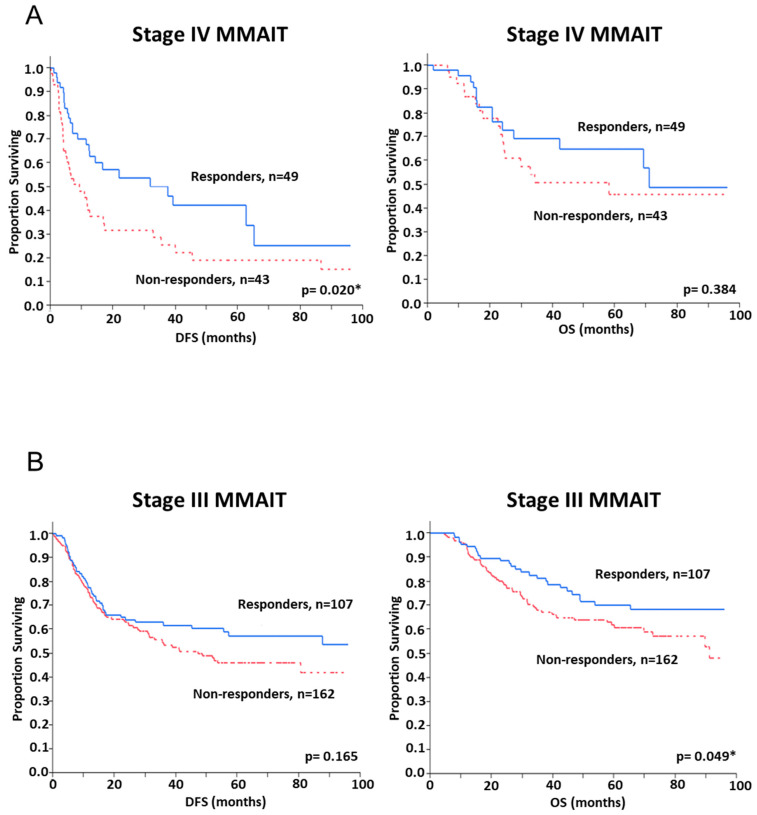
Kaplan Meier survival curves of patient groups defined by the gene SNP panel. Patients were classified into “responders” and “non-responders” by their SNP score found on the gene SNP panel. Kaplan-Meier survival curves were constructed for these two groups. (**A**) In the pilot cohort of stage IV MMAIT patients (*n* = 92), there was a significant difference in DFS between responders and non-responders (*p* = 0.020). (**B**) In the verification cohort of stage III MMAIT patients (*n* = 269), responders had an improved OS (*p* = 0.049). DFS = disease-free survival, OS = overall survival, MMAIT = Malignant Melanoma Active Immunotherapy Trial. * *p* < 0.05.

A Kaplan–Meier survival curve revealed a significant difference in DFS between responders and non-responders in the stage IV pilot cohort (*p* = 0.02) but no significant difference in OS was observed (Figure 5A). This was anticipated, as OS in Stage IV is typically short and less variable in advanced stage metastatic melanoma patients. As defined by the SNP panel analysis, patients in the responder group had a median DFS of 18.7 months compared to 9.4 months in the non-responder group. There was a trend observed towards an improved OS in the responder group when compared to the non-responder group, although statistical significance was not reached (Figure 5A). In multivariable Cox regression analysis, the biomarker panel SNP score was the only significant predictor of DFS with a hazard ratio (HR) of 1.84 (95% CI 1.09–3.13, *p* = 0.021) (Table 2). There were no significant associations between the SNP panel and patient clinicopathologic variables in this pilot cohort study. This pilot study demonstrated that the SNP panel score correlates to the DFS outcome only.

The SNP panel algorithm derived from the pilot cohort was then applied to the stage III MMAIT verification patient cohort. There were no significant associations between the SNP panel and patient clinicopathologic variables in the verification cohort. However, the SNP panel was able to classify the patients into responders versus non-responders with a significant difference observed in OS (*p* = 0.049), having a 75% survival rate at 46.3 months for responders versus 29.4 months for non-responders but no significant difference was observed in DFS between responders and non-responders (Figure 5B). The biomarker panel SNP score was also an independent predictor of OS (HR 1.67, 95% CI 1.07–2.67, *p* = 0.025) in multivariable Cox regression analysis (Table 3).

To confirm the predictive ability of a SNP panel to determine responders and non-responders to adjuvant BCG immunotherapy in melanoma patients, we assessed a control patient group of 80 AJCC stage III non-BCG or non-immunotherapy treated patients from our MSLT-I clinical trial [36,37,38]. We hypothesized that the immune SNP panel would not be an effective classification tool for patient outcome in this control patient cohort. The algorithm and classification method derived from the verification patient cohort was then applied to the stage III control cohort patients. There were no significant differences in DFS (*p* = 0.64) or OS (*p* = 0.93) in this control patient group, suggesting that the gene SNP panel applies to BCG immunotherapy responses and not to melanoma patients that are not receiving BCG therapy.

### 2.4. BCG Treated Immune SNP Panel Correlation to Patients’ DTH Response to PPD

In the MMAIT stage III multicenter clinical trial, patients (*n* = 269) were assessed for delayed type hypersensitivity (DTH) responses to determine their level of immune activation by BCG vaccination. To measure DTH responses, the purified protein derivative (PPD) skin test [26] was assessed on MMAIT clinical trial patients. PPD skin test response is used to assess the BCG innate immune activity in patients [39]. The level of DTH to PPDs quantification is described in the Materials and Methods section [39]. The analysis demonstrated a significant correlation between specific gene SNPs and DTH-PPD responses in BCG treated melanoma patients. The significantly correlated gene SNPs were: rs1143627 (IL1β), rs17215556 NRAMP1/SLC11A1, rs16944 (IL-1β) and rs1801157 (CXCL12) with *p* values of: *p* = 0.003, *p* = 0.029, *p* = 0.001, and *p* = 0.050, respectively (Table 4).

## 3. Discussion

The development of a predictive immune response biomarker to identify patients as potentially responsive or non-responsive to adjuvant immunotherapy is important for optimizing and triaging treatments for patients. The elucidation of factors for a patient’s clinical response to immune agents will allow for better selection of therapies with the greatest potential for success while minimizing the risks of ineffective therapies. Patients’ immunity is heterogeneous due to inherited genetic changes such as SNPs from specific key immune related genes. This is a critical problem in assessing human cancer immunotherapy responses. In mouse models, this inherited immune heterogeneity in treatment responsiveness is not as prevalent due to the genetic clonality of the inbred mouse models. In humans, inherited and innate immunity heterogeneity is quite significant and poorly understood in relation to patients’ cancer immunotherapy responses. Clinical studies focus on the immunotherapy agents’ direct efficacy and toxicity and not the hosts’ inherited immune system. Therefore, understanding the functional SNPs of specific genes during immunotherapy would be a beneficial predictive immunologic biomarker for a clinician to help select specific immunotherapies. This information could be used in addition to other known clinical factors in decision making. Our study demonstrates an essential neglected immune biomarker approach that can provide a new layer of importance in personalized cancer immunotherapy rationale to improve patient outcomes and treatment regimen decision making. Patients not likely to generate effective specific immune related responses due to inherited changes of specific cytokines/receptors, chemokines/receptors, and/or immune cell marker genes may not have a robust immune response to immunotherapies. Thus, alternative non-immunological based therapies may be more appropriate. This strategy can be applied to the usage of the modern day immunotherapies such as checkpoint inhibitor immunotherapies versus targeted therapies such as BRAF/MEK inhibitors for melanoma patients before treatment [1].

To address the issue of prospectively identifying patients that will respond to treatment from non-responders, we selected gene SNPs that represent a range of key immune pathways relevant to the activation of the BCG host innate immune system and melanoma anti-tumor immunity. Macrophages/monocytes play a key role in trained innate immunity, particularly in BCG responses, whereby they engulf the pathogen and stimulate lymphocytes and host immune responses [27]. These immune cells have also been implicated in controlling tumor growth and progression through tumor infiltration and promotion of inflammation. The release of these inflammatory molecules such as tumor necrosis factor (TNF) and specific cytokines can help mediate tumor apoptosis and control tumor cell proliferation [40]. In addition, macrophages/monocytes produce multiple pro-angiogenic factors, such as vascular endothelial factor (VEGF), granulocyte macrophage colony-stimulating factor (GM-CSF), IL-1, and IL-6, all of which are known to have significant effects on cancer cells [40]. BCG immunization is known to activate macrophage/monocytes in both cancer and non-cancer patients [13,27]. Specifically, NRAMP1 is expressed by macrophages and mediates innate resistance to the host infection by several pathogens, including BCG *Mycobacterium* [41,42,43]. We included several SNPs in the NRAMP1 gene that are reportedly associated with susceptibility to TB (tuberculosis) [44], autoimmune diseases [45], and a response to BCG therapy in patients with superficial bladder cancer [46]. In intravesical treatment of bladder cancer, BCG triggers a massive release of multiple types of cytokines and recruits immune cells, its therapeutic effect is associated with the induction of T helper type 1 and 2 (Th1/2) responses [13]. BCG is still considered an effective treatment for early stage bladder cancer [47], which implies that the activation of innate immune responses is effective in controlling cancer progression.

We also selected IL-1β for it is associated with enhanced trained innate immunity [48]. IL-1β is a pro-inflammatory cytokine, which is expressed by various immune cells including macrophages, and is known to play a critical role in innate host defense and helps stimulate the production of other inflammatory cytokines such as IL-8, IL-6, TNF-α, etc. [49,50]. Polymorphisms in inflammatory cytokines (TNF, IL-1β, IL-8, IL-10, IL-12β, and IL-23R) have been associated with TB and several inflammatory diseases [51,52,53,54]. IL-10 is associated with limiting resistance to infection against *M. tuberculosis* [55] and inhibiting Th1 immune response [56]. Dendritic cells are important antigen-presenting cells that, once activated, can present tumor-related antigens coordinating immune responses. DC-SIGN (dendritic cell-specific ICAM-3 grabbing non-integrin; CD209) is expressed on dendritic cells and functions in cell adhesion, pathogen recognition [57,58], and the uptake of a variety of pathogens including *Mycobacterium tuberculosis* [59].

TLRs are membrane receptors expressed by macrophages, T cells, and dendritic cells. SNPs in TLRs have been reported to confer susceptibility to various infectious and inflammatory diseases [60], including TB [61]. TLRs on immune and tumor cells have recently been shown to play an important role in the activation of immunotherapy [62]. TLR2 was selected due to its protective role and critical involvement in innate immune responses against mycobacteria [63,64].

Chemokines and chemokine receptors are known to regulate the phenotypic environment of tumors by influencing immune cell infiltration, tumor angiogenesis and survival, hence playing a critical role in tumor progression and therapeutic outcomes [65]. CCR5 and CXCL12 were selected due to their role in immune-response pathways in cancer patients [66,67]. Chemokine receptor CXCR4 and its ligand CXCL12 play an important homeostatic role in the homing and activation of macrophage lineage cell responses during infection and activating immune cells in tumor immune responses [68]. CCR5 polymorphisms have been shown to be associated with the survival of melanoma patients receiving immunotherapy [66,69]. CCR5 ligands are MIP (macrophage inflammation protein) α,β which attracts T cells. In contrast, CXCL12 is associated with tumor initiation and progression [67] and is involved in the recruitment of plasmacytoid dendritic cells (pDCs) [65].

SP110 is involved in the regulation of gene transcription. This protein has been reported to be linked to *Mycobacterium tuberculosis* resistance [34] and may influence cell apoptosis, differentiation and activation [70,71]. Several SP110 SNPs were found in populations with TB from West Africa, China, and India [70]. Lastly, BTNL2, which encodes a major histocompatibility complex, class II associated type I transmembrane protein belongs to the butyrophilin-like B7 family of immunoregulators. It is associated with decreased bacterial burden [72] and the downregulation of T cell activation [73,74].

DTH responses using the PPD test is a standard approach to measure BCG recall responses. DTH measures cell-mediated responses and the recruitment, activation, and stimulation of macrophages/monocytes, and T-cells to the site of challenging antigen injection. In a previous study, Faries et al. [26] showed that a positive DTH skin test is associated with increased survival in melanoma patients. In this study, we observed a significant correlation (*p* ≤ 0.05) between the DTH-PPD responses in BCG treated melanoma patients and the four gene SNPs from our panel: IL1β, NRAMP1/SLC11A1, and CXCL12. Involvement in the recruitment of immune cells, chemokines, and cytokines to the DTH challenge site are all important for a positive DTH-PPD response. Macrophages and IL-1β secretion are known to be involved in the DTH responses [75,76]. CXCL12 is a chemokine that attracts CXCR4+ immune cells, such as macrophage/monocytes, T cells, and pDCs [65,68]. This demonstrated an in vivo functional relation of DTH related to BCG recall immunity involving specific immune SNP genes.

Using the established SNP gene coefficient formula and cut-off point initially derived from more advanced melanoma patients (MMAIT Stage IV, pilot cohort), we developed a 26 gene panel. We then applied this panel to stage III MMAIT multicenter clinical trial melanoma patients who benefited from BCG treatment with an improved disease outcome. The use of gene SNPs potentially allows for the identification of patients rendered disease-free who will likely have a poor disease outcome. This suggests patients who had sub-clinical systemic disease can have effective tumor immune control through innate immune system priming, by BCG. We have verified a novel gene panel of 26 SNPs effectiveness in predicting disease outcome after adjuvant BCG therapy. This SNP panel requires further verification in a larger adjuvant clinical trial. It is interesting that BCG treatment is effective in early-stage melanoma patients with a subclinical tumor burden very similar in its effectiveness to treating early-stage bladder cancer.

This panel may be applied to other immunotherapies in melanoma patients. New gene SNPs can be developed to address modern immunotherapy agents such as anti-CTLA-4, PDL-1,PD-1, and cancer vaccines. It is inevitable that individual immunotherapies activating specific types of immune responses can have different gene SNPs. If subsequent validation studies are successful, the SNP panel may be included as an important tool, useful prior to the selection of immunotherapy for patients.

Previous studies reported that BCG provides protection against multiple microorganisms such as bacteria (*S. aureus*), fungi (*C. albicans*), and viruses (yellow fever virus) [77]. Moreover, a recent clinical trial of BCG vaccination showed that it can protect the elderly against respiratory tract infections [78]. Innate immune cells can be “primed” by BCG, which is known as trained innate immunity that can be effective against pathogen infections and early stage tumors [79]. It is also said to be involved in the transcriptional and epigenetic rewiring of innate immune cells (e.g., myeloid and NK cells), which leads to an increase in cytokine production and an enhanced killing capacity [27,78,80]. Additionally, BCG can provide a long-lasting change in the immune system specifically in heterologous T helper 1 (Th1) and Th17 immune responses in innate trained immunity [81]. With the ongoing COVID-19 pandemic, there have been reports that patients who are BCG-vaccinated are less susceptible to serious illness and have less severe COVID-19 symptoms [82]. Several studies are currently being conducted worldwide to determine whether BCG vaccination has a protective effect against COVID-19 (clinicaltrials.gov#–NCT04537663, NCT04379336, NCT04369794, NCT04461379, NCT04347876) [82,83,84,85]. These studies indicate that the immunologic SNP panel could also be utilized as an important tool if BCG is preventative or can reduce the pathogenicity of COVID-19 as well as other infectious diseases.

## 4. Materials and Methods

### 4.1. Melanoma Patients

This study involved AJCC stage III and IV melanoma patients from the multicenter phase III clinical trials. These patients were divided into 3 cohorts: pilot, verification, and control. All patients were entered into the multicenter phase III clinical trials, had undergone complete surgical resection and were rendered free of primary and metastatic disease [16,17]. Post-operative computed tomography (CT) of the chest, abdomen, and pelvis, and magnetic resonance imaging (MRI) or CT of the brain was used to confirm no evidence of disease prior to the initiation of adjuvant therapy or follow-up without treatment. Patients in the pilot Stage IV patients (*n* = 92) and verification Stage III patient (*n* = 269) cohorts were randomized to receive BCG adjuvant immunotherapy after being rendered disease-free by surgery, and were enrolled in the phase III international Malignant Melanoma Active Immunotherapy Trials (MMAIT) Stage IV and Stage III, respectively, sponsored by JWCI trial center [see references for complete details of the trials] [16,17,26]. Patients in the control cohort did not receive BCG or any form of immunotherapy and were enrolled in the phase III international study, Multicenter Selective Lymphadenectomy Trial (MSLT-I). MSLT-I patients assessed in this control cohort were SLN (+) and received complete lymph node dissection to be rendered disease-free status [36,37,38,86].

The MMAIT studies were two phase III randomized double-blind international multicenter trials comparing BCG plus a melanoma cell vaccine (CanVaxin^TM^) versus BCG plus placebo after complete surgical resection rendering patients disease-free in AJCC stage III and IV melanoma patients (clinicaltrials.gov# NCT00052130, stage III study; #NCT00052156, stage IV study) (Figure 1 CONSORT diagram) [16,17,26]. Only the BCG treatment plus placebo arm was assessed. The placebo given was RPMI-1640 containing 7.5% human serum albumin and 10% dimethyl sulfoxide. The studies enrolled participants between May 1998 and March 2005, with study follow-up until May 2010 (Figure 1 and Figure 2). Eligible participants were patients 18–80 years of age, with histologically confirmed metastatic melanoma (AJCC stage III or IV), clinically disease-free after complete lymphadenectomy or metastasectomy, and Eastern Cooperative Oncology Group (ECOG) performance status of 0 or 1. There were 1656 patients (1160 AJCC stage III patients and 496 AJCC stage IV patients) enrolled in the MMAIT studies. BCG (Tice strain; Organon Technika, Durham, NC, USA) was used as an adjuvant immunotherapy. CanVaxin^TM^ is an allogeneic whole-melanoma cell vaccine (3 irradiated characterized melanoma cell lines) [87]. Following randomization and within 90 days of complete surgical resection rendering them disease-free, participants were treated with BCG at days 0 and 14. BCG doses were 3 × 10^6^ colony forming units (CFU) on day 0 and 1.5 × 10^6^ CFU on day 14. Patients in the BCG plus CanVaxin^TM^ group received CanVaxin^TM^ at days 0, 14, 28, 42, 56, and then monthly thereafter during year 1, every 2 months in year 2, and every 3 months in years 3–5 (Figure 2). Participants in the BCG plus placebo group received injections at these same time periods. BCG was administered intradermally in the axilla or groin area on days 0 and 14. Patient peripheral blood leukocytes (PBLs) were procured from heparinized blood drawn before BCG treatment, processed and cryopreserved at −80 °C in DMSO medium as previously described [17]. The Institutional Review Board (IRB) at JWCI/Saint John’s Health Center and all participating clinical trial sites have approved the clinical trial and companion biomarker study [17]. This study followed the principles in the Declaration of Helsinki. All participants provided IRB approved informed written consent.

Patients in the control cohort (no BCG or other immunotherapy treatment) were enrolled from the MSLT-I clinical trial (clinicaltrials.gov# NCT00275496) (Figure 1 CONSORT diagram) [36,37]. MSLT-I is a phase III international multicenter clinical trial that randomized melanoma patients with invasive primary melanoma with no clinical evidence of lymph node metastasis. Patients underwent wide skin excision and SLN biopsy, followed by complete lymphadenectomy for resection of potential metastases versus wide excision and regional draining nodal observation with subsequent lymphadenectomy for nodal recurrence during observation. Patients in MSLT-1 were enrolled between 20 January 1994 and 29 March 2002 (10-year follow-up available for MSLT-I). Patients used in the MSLT-I trial had pathology defined metastasis and were classified as AJCC Stage III.

### 4.2. DTH-PPD Test

Delayed type hypersensitivity (DTH) to specific antigens is widely used as a measure of host immune competence [39]. DTH to BCG was assessed by the purified protein derivative (PPD) test in the MMAIT-III patients prior to the first injection of BCG plus placebo and at day 28, day 56, and month 4, or until positive. This standard assay is commonly used in an in vivo skin test to determine levels of response to BCG by applying an intradermal injection of tuberculin (PPD) to a patient’s forearm and then reading the reaction between 48–72 h after administration [26]. After 48 h, DTH responses were assessed and measured in mm, and a positive cut-off at ≥7 mm was applied [39]. The largest DTH response within 4 months after the patient entered the trial was used for analysis [39].

### 4.3. Peripheral Blood Leukocytes (PBLs) Collection

Peripheral blood was processed for leukocytes and cryopreserved in liquid nitrogen as previously described [17]. Due to logistics, processing for PBL specimens was carried out at U.S. cancer center sites only. Thus, only patients in the BCG plus placebo group from U.S. centers were enrolled in the MMAIT studies. Results in the pilot cohort of patients with stage IV melanoma in the MMAIT-IV study were verified using the verification cohort of patients with AJCC stage III melanoma who were selected from the MMAIT-III study. PBLs were collected prior to surgery similar to the MSLT-I trial.

### 4.4. Experimental Approach

Candidate SNPs were identified by a literature review of genetic polymorphisms associated with macrophage cytokines or monocyte-related immune response pathways to BCG and/or TB. The final biomarker panel in this study consisted of 26 SNPs as presented in Table 1 and can be broadly grouped into immune related genes: macrophages/monocytes (NRAMP1/SLC11A1, CD14, CD18); dendritic cells (CD209/DC-SIGN); TLR (TLR2); inflammatory cytokines (TNF, IL1β, IL8, IL10, IL12β, and IL23R); and chemokine/chemokine receptors (CCR5, CXCL12).

For the pilot, verification, and control patient cohorts, we extracted DNA from cryopreserved PBLs by QIAamp DNA mini kit (QIAGEN, Valencia, CA, USA) as previously described [88]. For the control cohort, DNA was extracted from Flinders Technology Associates (FTA) cards (VWR International, Radnor, PA, USA), which were spotted with whole blood using the GenSolve kit (GenVault, Carlsbad, CA, USA). DNA was quantified using the Quant-iT PicoGreen dsDNA assay kit (Invitrogen, Carlsbad, CA, USA) as previously described [89]. DNA was characterized for the candidate SNPs by MassARRAY MALDI-TOF Mass Spectrometry (Sequenom, San Diego, CA, USA) [89]. PCR and iPLEX (extension) primers were designed by using SEQUENOM database. Data analysis was performed with MassARRAY Typer 4.0.20 software, as previously described [90]. If peaks of the specific gene SNP were not well defined, they were repeated until clear. If clarity was not significant, the patient sample data was not used.

### 4.5. Biostatistical Analysis

The primary endpoints were overall survival (OS) and disease-free survival (DFS). OS was defined as time to death or censored if alive at the time of the last follow-up in 2010. DFS was defined as the time to melanoma recurrence, death, or censored if without melanoma recurrence at the date of the last follow-up. Survival time was calculated from the date of randomization to the date of event (death or melanoma recurrence) or the last follow-up.

We chose to analyze the genotype data by Principal Component Analysis (PCA) [91]. This method has been used commonly for visualization and analysis of high dimensional data characteristic of multi-gene genomic studies. The large number of SNPs evaluated allowed us to avoid using models that fit parameters to outcome (e.g., proportional hazards models) overfitting.

The allele status of the 26 SNPs was determined for each patient as described in the experimental approach. We considered the chromosomal status (i.e., common homozygotes, heterozygotes, and rare homozygotes) at each SNP as continuous variables, assigning one point for each allele [92]. For example, for a SNP with an allele “A”, if the patient’s genotype for that SNP was “AA” then 2 is assigned; the value is 1 if the genotype is AG; and if the genotype is GG then the value is 0. This was carried out for all 26 SNPs, and PCA was conducted to determine the coefficient for each SNP. Principal component 2 (PC2) was the first to show a dichotomized distribution. PC2 will be referred to as the patient’s “SNP score”, which is calculated using PC2 = P_1_X_1_ + P_2_X_2_ + … + P_27_X_27_, where P_i_ is the principal component 2 coefficient for each SNP as generated by the PCA algorithm, while X_i_ is each individual SNP’s allelic state as a value of 0, 1, or 2, which is determined as outlined above. From our PCA data, a cut-off of 0.005 was selected from the mean (and by visual inspection) of component 2 from 92 patients in the pilot cohort (MMAIT stage IV BCG-treated patients), and they were separated into two groups, termed here as responsive (Comp2 ≤ 0.005) and non-responsive (Comp2 > 0.005) to adjuvant BCG therapy.

We hypothesized that patients at later stages of disease would acquire mutations and prognostic markers correlating with poor disease outcome. These markers could then be applied to earlier stages of the disease to predict patients’ outcome at an earlier stage. PCA was applied to the pilot cohort from whom a SNP coefficient formula and cut-off point were derived (Figure 4). The same SNP coefficient formula and cut-off point were then applied to the verification and control cohorts.

Associations between SNP status and clinicopathologic factors were assessed by Pearson’s correlation coefficient for continuous variables and Student’s T-test or ANOVA for categorical variables. We analyzed survival by the Kaplan-Meier method. Multivariable survival analyses by stepwise Cox proportional hazards analysis were carried out using known prognostic covariates (i.e., age, sex, Breslow thickness, site of primary lesion, ulceration, and number of positive lymph nodes). All *p*-values were two-sided and *p* ≤ 0.05 was considered statistically significant. All statistical analyses were performed with JMP^®^, Version 9.0 (SAS Institute, Inc., Cary, NC, USA). The significant correlation between the SNP panel to DTH-PPD responses in BCG treated melanoma patients was assessed by performing the likelihood ratio and Chi-Square test.

## 5. Conclusions

This study is the first immune SNP-based predictive multi-biomarker test for adjuvant BCG immunotherapy in AJCC stage III and IV metastatic melanoma patients in a multicenter clinical trial setting. In this study, we successfully determined the allelic status of the 26 SNPs for each patient in all three melanoma cohorts (AJCC stage III, stage IV, and control Stage III) and categorized them into two outcome groups: responders and non-responders to adjuvant BCG therapy. As a result, by using the 26 SNP panel we were able to demonstrate that responders to adjuvant BCG therapy showed a significant improvement in DFS in the pilot cohort of stage IV melanoma patients and OS in the verification cohort of stage III melanoma patients.

## Figures and Tables

**Table 1:** Genomic biomarker panel of 26 SNPs and their allelic states.

SNP	Gene	Chromosome	Position GRCh37	Minor Allele Frequency (dbSNP)
Macrophage				
rs17215556	SLC11A1/NRAMP	2	219,258,856	0.0060 (T > C)
rs17235409	SLC11A1/NRAMP	2	219,259,732	0.0702 (G > A)
rs17235416	SLC11A1/NRAMP	2	219,259,814	0.1024 (del TGTG)
rs34448891	SLC11A1/NRAMP	2	219,246,649	MAF not reported (ins GT)
rs3731865	SLC11A1/NRAMP	2	219,250,003	0.1680 (G > C)
rs2569190	CD14	5	140,012,916	0.4734 (A > G)
rs2569193	CD14	5	140,015,495	0.2172 (G > A)
rs684	CD18	21	46,306,161	0.1639 (G > A)
rs117989670	CD18	21	46,305,913	0.0069 (A > C)
Dendritic cells				
rs4804803	CD209	19	7,812,733	0.2117 (A > G)
Toll-like receptor				
rs4696480	TLR2	4	154,607,126	0.4601 (T > A)
rs5743708	TLR2	4	154,626,317	0.0119 (G > A)
Inflammatory cytokines				
rs1800629	TNF	6	31,543,031	0.0955 (G > A)
rs361525	TNF	6	31,543,101	0.0505 (G > A)
rs1143627	IL1B	2	113,594,387	0.4803 (C > T)
rs1143634	IL1B	2	113,590,390	0.1455 (C > T)
rs16944	IL1B	2	113,594,867	0.4651 (A > G)
rs4073	IL8	4	74,606,024	0.4972 (A > T)
rs1800896	IL10	1	206,946,897	0.3026 (A > G)
rs17860508	IL12B-near 5′	5	158,760,200	not reported
rs1343151	IL23R	1	67,719,129	0.3237 (G > A)
Chemokines/Chemokine receptor				
rs1799987	CCR5	3	46,411,935	0.4871 (A > G)
rs1801157	CXCL12	10	44,868,257	0.2080 (G > A)
Others				
rs3948464	SP110	2	231,050,715	0.1028 (C > T)
rs1135791	SP110	2	231,042,276	0.3375 (T > C)
rs2076530	BTNL2	6	32,363,816	0.3779 (A > G)

**Table 2:** Multivariable stepwise Cox regression analysis of disease outcome: biomarker SNP panel and clinicopathologic characteristics in the discovery cohort of MMAIT stage IV BCG-treated patients.

Variable	Disease-Free Survival		Overall Survival	
	Multivariable Analysis		Multivariable Analysis	
	* *p*-Value	HR (95% CI)	* *p*-Value	HR (95% CI)
Immune-gene Panel SNP Score (Responder vs. Non-Responder)	0.021	1.84 (1.09–3.13)		
Age				
Gender				
Male/Female				
Lymph Node Positive				
1				
0				
2–3				
≥4				
Unknown				
Primary Site				
Extremity				
Head/Neck				
Mucosal				
Trunk				
Unknown				
M-Stage				
M1a				
M1b				
M1c				
Baseline LDH				
Normal /Elevated				
Number of Metastasis				
1				
2–3				
4–5				
ECOG Performance Status				
0				
1				
Unknown				
Prior Diagnosis of Stage III Melanoma				
Yes/No				
Previous Treatment for Melanoma				
Yes/No				

* Variables that do not have a *p*-value shown were not significant. Bold font indicates *p* value ≤ 0.05.

**Table 3:** Multivariable stepwise Cox regression analysis of survival: biomarker SNP panel and clinicopathologic characteristics in the validation cohort of MMAIT stage III BCG-treated patients.

Variable	Disease-Free Survival		Overall Survival	
	Multivariate Analysis		Multivariate Analysis	
	* *p*-Value	HR (95% CI)	* *p*-Value	HR (95% CI)
Immune-gene Panel SNP Score (Responder vs. Non-Responder)	0.158	1.31 (0.90–1.93)	**0.025**	1.67 (1.07–2.67)
Age	**0.007**	1.02 (1.01–1.03)		
Gender			**0.040**	1.64 (1.02–2.71)
Male/Female				
Breslow				
≤1.00 mm		1.00 (Reference)		
1.01–2.00 mm		0.71 (0.40–1.30)		
2.01–4.00 mm		0.59 (0.32–1.11)		
>4.00 mm		0.98 (0.52–1.85)		
Unknown		0.55 (0.29–1.04)		
Lymph Node Positive			**0.048**	
1				1.00 (Reference)
2–3			**0.014**	1.81 (1.13–2.89)
≥4				1.31 (0.67–2.44)
Primary Site				
Extremity				
Head/Neck				
Mucosal				
Trunk				
Unknown				
Palpable Lymph Node				
Yes/No		1.33 (0.90–1.97)	**0.029**	1.68 (1.05–2.69)
Ulceration			**0.016**	
No		1.00 (Reference)		1.00 (Reference)
Yes	0.061	1.60 (0.98–2.63)	**0.008**	2.08 (1.21–3.65)
Unknown		1.02 (0.64–1.65)		1.13 (0.65–2.00)

* Variables that do not have a *p*-value shown were not significant. Bold font indicates *p* value ≤ 0.05.

**Table 4:** BCG treated MMAIT stage III melanoma patient cutaneous DTH response to PPD correlation to immune gene SNP panel.

Gene	SNP	*p* Value
IL1β	rs1143627	0.003
NRAMP1/SLC11A1	rs17215556	0.029
IL-1β	rs16944	0.001
CXCL12	rs1801157	0.050

All other gene SNPs did not significantly correlate to DTH-PPD response. *p* value was assessed by performing the likelihood ratio chi-square test (*n* = 269 patients).

## Data Availability

Data is contained within the article or Supplementary Materials.

## Supplementary Materials

The following are available online at https://www.mdpi.com/2072-6694/13/1/91/s1, Figure S1. Additional principal component analysis (PCA) of immune gene SNP panel in the pilot patient cohort (MMAIT stage IV BCG-treated). Table S1A: Clinicopathologic characteristics of study patients: Pilot cohort of MMAIT stage IV BCG+Placebo-treated patients (*n* = 92). Table S1B: Clinicopathologic characteristics of study patients: Verification cohort of MMAIT stage III BCG-treated patients (*n* = 269). Table S1C: Clinicopathologic characteristics of study patients: Control cohort of MSLT-1 stage III non-BCG and non-immunotherapy treated patients (*n* = 80).

## Abbreviations

AJCC	American Joint Committee on Cancer
SNP	Single Nucleotide Polymorphism
BCG	Bacillus Calmette–Guérin
SLN	Sentinel lymph node
DFS	Disease free survival
HR	Hazard ratio
OS	Overall survival
IL	Interleukin
ICI	Immune Checkpoint Inhibitors
GM-CSF	Granulocyte-macrophage colony-stimulating factor
TLR	Toll-Like Receptor
MSLT-I	Multicenter Selective Lymphadectomy
MMAIT	Malignant Melanoma Active Immunotherapy Trial
TNF	Tumor necrosis factor
CCR5	C-C chemokine receptor type 5
CXCL12	C-X-C motif chemokine 12
NRAMP1	natural resistance-associated macrophage protein 1
SLC11A1	solute carrier protein 11A1
CD209	Cluster of Differentiation 209
DC-SIGN	Dendritic Cell-Specific Intercellular adhesion molecule-3-Grabbing Non-integrin
BTNL2	Butyrophilin Like 2
PCA	Principal Component Analysis
DTH	Delayed Type Hypersensitivity
PPD	Purified Protein Derivative
VEGF	Vascular Endothelial Factor
pDC	Plasmacytoid Dendritic Cells
CT	Computed Tomography
MRI	Magnetic Resonance Imaging
ECOG	Eastern Cooperative Oncology Group
PBL	Peripheral Blood Leukocytes
IRB	Institutional Review Board
DTH	Delayed Type Hypersensitivity
MIP	Macrophage Inflammation Protein
TB	Tuberculosis
ECOG	Eastern Cooperative Oncology Group
CLND	Complete Lymph Node Dissection
