# Supplementary material for: Genetic Variants in Immune Related Genes as Predictors of Responsiveness to BCG Immunotherapy in Metastatic Melanoma Patients
Source: Cancers (Basel). 2020 Dec 30;13(1):91. doi: 10.3390/cancers13010091 (PMC7795941; doi:10.3390/cancers13010091)
Supplement: Supplementary file 1 [file cancers-13-00091-s001.pdf]

Figure S1

A

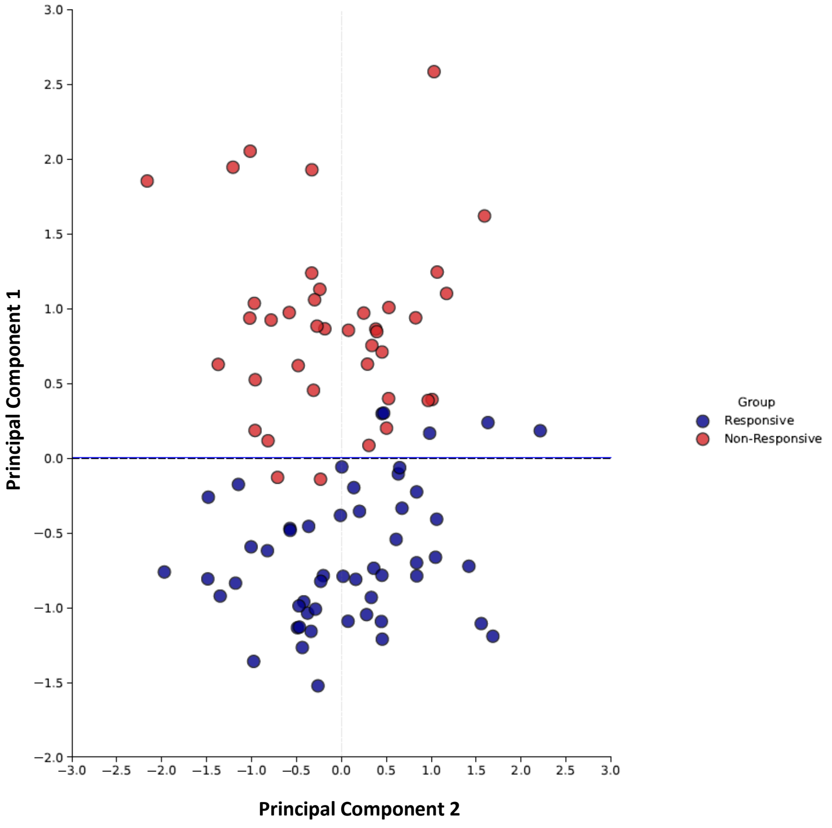

B

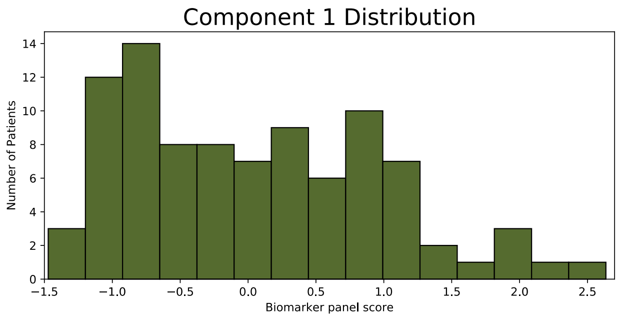

C

SNP	Principal Component 1
rs1135791-C	0.061898905
rs1143627-T	–0.572914865
rs1143634-C	0.223968507
rs1343151-C	0.181084147
rs16944-C	–0.572914865
rs17215556-C	–0.001129828
rs17235409-G	–0.035938289
rs17235416-TGTG	–0.035938289
rs17860508-GC	–0.01106161
rs1799987-A	0.378582413
rs1800629-G	0.065297729
rs1800896-A	0.036832864
rs1801157-G	0.068161602
rs2076530-A	0.026778846
rs2569190-G	0.189771597
rs2569193-A	–0.017513235
rs34448891-GT	1.05879E-22
rs361525-G	–0.038789993
rs3731865-C	0.102320583
rs3948464-C	–0.020225295
rs4073-A	0.197724616
rs4696480-T	0.044585597
rs4804803-A	0.039180265
rs5743708-G	0.037086666
3UTR122GA-A	0.082013453
3UTR370GT-G	–0.01773406

Figure S1. Additional principle component analysis (PCA) of immune gene SNP panel in the pilot patient cohort (MMAIT stage IV BCG-treated). (A) The immune gene panel consisting of the 26 common and relevant SNPs with their respective scores for each patient are represented as data points on the PCA plot. The first and second principal components represent the most variation among the SNP scores. Non-responders show lower values for the first principal component. The first component therefore separates responders from non-responders based on their SNP scores. Grey line = cut off point at 0.005. Responders: Comp2 ≤ 0.005; Non-responders: Comp2 > 0.005. **(B)** Histogram showing the distribution of principal component 1 among all patients. **(C)** Co-efficient for each SNP in principal component 1 calculated by the PCA.

Table S1A. Clinicopathologic characteristics of study patients: Pilot cohort of MMAIT stage IV BCG+Placebo-treated patients (n=92)

Clinicopathologic Variable		Number of Patients (%)
Age (Years)*		55.4 ± 11.8
Gender		
	Male	54 (59%)
	Female	38 (41%)
Lymph Node Positive		
	0	3 (3%)
	1	13 (14%)
	2-3	6 (7%)
	≥4	31 (34%)
	Unknown	39 (42%)
Breslow		
	≤1.00mm	20 (22%)
	1.01-2.00mm	22 (24%)
	2.01-4.00mm	13 (14%)
	>4.00mm	11 (12%)
	Unknown	26 (28%)
Primary Site		
	Extremity	32 (35%)
	Head/Neck	18 (20%)
	Trunk	21 (23%)
	Mucosal	1 (1%)
	Unknown	20 (22%)
M-Stage		
	M1a	27 (29%)
	M1b	20 (22%)
	M1c	45 (49%)
Baseline LDH		
	Normal	40 (43%)
	Elevated	52 (57%)
Number of Metastasis		
	1	48 (52%)
	2-3	41 (45%)
	4-5	3 (3%)
ECOG Performance Status		
	0	77 (84%)
	1	14 (15%)
	Unknown	1 (1%)
Prior Diagnosis of Stage III Melanoma		
	Yes	52 (57%)
	No	40 (43%)
Previous Treatment for Melanoma		
	Yes	41 (45%)
	No	51 (55%)

* Average ± 1 Standard Deviation

Table S1B. Clinicopathologic characteristics of study patients: Verification cohort of MMAIT stage III BCG-treated patients (n=269)

Clinicopathologic Variable		Number of Patients (%)
Age (Years)*		52.7 ± 13.8
Gender		
	Male	165 (61%)
	Female	104 (39%)
Lymph Node Positive		
	1	151 (56%)
	2-3	82 (30%)
	≥4	36 (13%)
Breslow		
	≤1.00mm	43 (16%)
	1.01-2.00mm	67 (25%)
	2.01-4.00mm	63 (23%)
	>4.00mm	47 (17%)
	Unknown	49 (18%)
Primary Site		
	Extremity	103 (38%)
	Head/Neck	32 (12%)
	Trunk	97 (36%)
	Mucosal	2 (1%)
	Unknown	35 (13%)
Ulceration		
	Yes	74 (28%)
	No	90 (33%)
	Unknown	104 (39%)
Palpable Lymph Node		
	Yes	123 (46%)
	No	146 (54%)

* Average ± 1 Standard Deviation

Table S1C. Clinicopathologic characteristics of study patients: Control cohort of MSLT-1 stage III non-BCG and non-immunotherapy treated patients (n=80)

**Clinicopathologic Variable**		**Number of Patients (%)**
Age (Years)*		50.4 ± 13.8
Gender		
	Male	49 (61%)
	Female	31 (39%)
Lymph Node Positive		
	1	67 (84%)
	2-3	12 (15%)
	≥4	1 (1%)
Breslow		
	≤1.00mm	5 (6%)
	1.01-2.00mm	22 (28%)
	2.01-4.00mm	40 (50%)
	>4.00mm	13 (16%)
Primary Site		
	Extremity	31 (39%)
	Head/Neck	16 (20%)
	Trunk	33 (41%)
Ulceration		
	Yes	33 (41%)
	No	47 (59%)

* Average ± 1 Standard Deviation
